# Editorial: Technologies for diabetes

**DOI:** 10.3389/fendo.2023.1198029

**Published:** 2023-05-04

**Authors:** Giuseppina Salzano, Davide Tinti, Roque Cardona-Hernandez, Maurizio Delvecchio

**Affiliations:** ^1^ Department of Human Pathology in Adult and Developmental Age “Gaetano Barresi”, University of Messina, Messina, Italy; ^2^ Department of Public Health and Paediatric Sciences, University of Turin-Regina Margherita Children Hospital-Azienda Ospedaliero-Universitaria (A.O.U.) Città della Salute e della Scienza di Torino, Turin, Italy; ^3^ Division of Pediatric Endocrinology, Hospital Sant Joan de Déu, Barcelona, Spain; ^4^ Metabolic Disorders and Diabetes Unit, “Giovanni XXIII” Children’s Hospital, Azienda Ospedaliero-Universitaria (AOU) Policlinico-Giovanni XXIII, Bari, Italy

**Keywords:** diabetes mellitus, technology, hybrid closed loop system, advanced hybrid closed loop system, flash glucose monitor, quality of life, MiniMed 780G, tandem control-IQ

Much progress has been made in technologies for diabetes mellitus over the last decade. Continuous glucose monitoring (CGM) and flash glucose monitoring (FGM) systems have achieved high accuracy and reliability, yielding a large use worldwide. The development of progressively smarter closed-loop systems, which combine insulin pumps and glucose monitoring systems allows for the minimization of hypo- and hyperglycemia through automatic insulin delivery. This Research Topic encloses high-quality manuscripts on the topic.

In her review, Templer offers an interesting update about the closed-loop systems used in the treatment of type 1 diabetes. She traces the rapid progress from the past and their use in the present, focusing attention on what future therapeutic strategies will be, including fully closed-loop systems. Questions regarding faster-acting insulin or the addition of other hormones (such as glucagon) to mitigate the risk of hypoglycemia or more advanced algorithms are debated. She concludes that the answers lay in the next generation of closed-loop therapy, which will probably use a combination of different therapeutic options. The step from hybrid closed loop (HCL) to advanced HCL (AHCL) allows an improvement in blood glucose control which is shown by real-world studies.

Flash glucose monitoring (FGM) is a novel device type capable of showing interstitial glucose and trends on a reader in an on-demand fashion, with good reproducibility and similar value to blood glucose measurements (1). The use of FGM is associated with better glucose outcomes in adults with type 1 diabetes (2) but is effective in reducing HbA1c and glucose variability in children and adolescents as well (3). The key role of education in properly using these technologies is highlighted by Lee et al. They demonstrated that personalized and continuous education may significantly improve blood glucose control in adult patients.

FGM and its interpretation have been shown to be effective also in the management of physical activities, using glucose values and trends to adapt therapy before, during, or after exercise (4). In the study by Guo et al., benefits around the system, as well as proper usage, have been shown to be related to actually watching the glucose values, while a blinded use resulted in similar values to usual care. Furthermore, Hohendorff et al. showed through the Hypoglycemia Fear Survey II questionnaire that the use of FGM is associated also with less fear of hypoglycemia, reducing diabetes distress. On the other hand, Franceschi et al. showed that the early use of FGM (within the first month after type 1 diabetes onset) plays an important role in metabolic control and quality of life in children and adolescents. In this real-world study, the authors showed a reduction of HbA1c during the first year and interestingly a longer partial remission phase in the group of patients with early use of FGM compared to the control group. This Research Topic also includes a paper on the effectiveness of FGM in adults with type 2 diabetes on premixed insulin therapy by Yan et al. They showed that real-time FGM improves blood glucose control and diabetes self-care better than retrospective FGM.

Diabetes is the main cause of chronic kidney disease (CKD), and it is mandatory to achieve optimal glycemic control with the aim to reduce the risk of progression of CKD and related death. It has been recently described that the use of CGM is recommended in patients with advanced CKD (5), but unfortunately, data are lacking in this population. Ling et al. review literature data and show that HbA1c and alternative glycemic markers have limitations in patients with advanced CKD, and thanks to CGM-derived glucose management indicator (GMI), it is now possible to monitor the glycemic status with better precision in these patients.

Using last-generation systems to time AHCL, an improvement in main glucose outcomes (namely time below –TBR-, in –TIR-, and above range - TAR) compared to Sensor Augmented Pump (SAP) therapy in a population of patients with type 1 diabetes has been demonstrated. Control-IQ (CIQ) system is one of the algorithms recently approved for children and adults with diabetes (6). The group by Bassi et al. published three papers about the effectiveness of new algorithms in blood glucose management. In the first paper, they investigated the effectiveness of a new function of CIQ in the improvements of glucose values during nighttime (Sleep Activity). They showed that it seems to be less effective than the standard CIQ algorithm in terms of TIR. In a head-to-head study, they compare two AHCL systems, Tandem t:slim X2 Control IQ™ system (Tandem Inc., San Diego, California) and the Minimed™ 780G system (Minimed Medtronic, Northridge, California), in 90 patients (aged 5 to 65 years) with type 1 diabetes enrolled in a retrospective dual-center study. On the basis of their results, the authors report that the Minimed 780G system seems more effective in managing hyperglycemia, while Tandem Control-IQ reduces the number of hypoglycemic episodes and glucose variability and that both systems achieve the recommended glycemic targets (Bassi et al.). On the other hand, the same authors show in their single-center study that after 1 year of use, the CIQ system allowed a TIR of 68%, which is significantly lower than the MiniMed 780G (71%) (Bassi et al.).

In type 1 diabetes pediatric real-world settings, a superiority of HCL systems versus other technologies as demonstrated by higher levels of time spent in the target glucose range and the reduction of both hypoglycemic and hyperglycemic events over a 1-year period (7) is reported.

Recently, Lombardo et al. (8) conducted a multicenter observational real-world 6-month study with the aim to investigate glycemic outcomes in a large cohort of children and adolescents with type 1 diabetes over the first 6-month use of MiniMed™ 780G, and the study shows that the most relevant targets are achieved according to International Consensus. Both at 3 and 6 months, 39.6% of participants reached all the glycemic targets (TIR, CV, GMI, and TBR). Authors also reported that older age, shorter disease duration, and shorter active insulin time are significant predictors of optimal glucose control (8).

The effectiveness of a SAP with predictive low glucose suspend (SmartGuard™) versus a pump with independent FGM (Freestyle libre^®^) has been investigated in 6 to 14 years old children with type 1 diabetes. No significant difference in blood glucose control is reported among the two groups, but the decision of all families to continue with CGM after the study suggests that this system has a positive impact on diabetes burden, preferring the SmartGuard^®^ system (Schierloh et al.).

Technologies may also reveal how patients manage special events in their daily life. Molveau et al. investigated the impact of daily physical activity on nocturnal hypoglycemia through a blinded CGM. They concluded that patients do not properly report insulin boluses and compensation strategies, suggesting that appropriate education is still needed in such situations. CGM may be used also to investigate blood glucose control in the diagnostic work-up. In their paper, Zhang et al. compared CGM metrics between patients with type 2 diabetes and latent autoimmune diabetes (LADA). Interestingly, they showed that patients with LADA presented wider glucose variation and thus they suggested that data from CGM could be helpful for the diagnostic work-up in a patient with glucose control impairment.

Technologies have backlashes as well and dermatological complication is one of the most frequent. Skin exposure to chemical and mechanical agents may lead to skin disorders and, overall, to contact dermatitis. In their observational study, Passanisi et al. described the clinical impact of this specific complication, providing helpful information for clinicians about the current management and the possible effect of such problems.

Technologies for diabetes are a growing field of research and represent a great promise for patients with diabetes. We would like to end this Editorial by focusing the readers’ attention on relevant data for clinical practice. Over the last 5 years, we passed from the HCL system (MiniMed 670G^®^), which allowed a TIR of approximately 65 to 70% and a TBR below 4% (9), to the new AHCL systems which allow a TIR of approximately 75% and a further reduction in TBR (10, 11). Blood glucose control improves as fast as technologies for diabetes go on.

## Author contributions

All authors listed, have made substantial, direct, and intellectual contribution to the work, and approved it for publication.

## References

[1] Moser O Sternad C Eckstein ML Szadkowska A Michalak A Mader JK. Performance of intermittently scanned continuous glucose monitoring (isCGM) systems in people with type 1 diabetes: a pooled analysis. Diab Obes Metab (2022) 24(3):522–9. doi: 10.1111/dom.14609 34866293

[2] Bruttomesso D Laviola L Avogaro A Bonora E Del Prato S Frontoni S. The use of real time continuous glucose monitoring or flash glucose monitoring in the management of diabetes: a consensus view of Italian diabetes experts using the Delphi method. Nutr Metab Cardiovasc Dis (2019) 29(5):421–31. doi: 10.1016/j.numecd.2019.01.018 30952574

[3] Zhang W Liu Y Sun B Shen Y Li M Peng L. Improved HbA1c and reduced glycaemic variability after 1-year intermittent use of flash glucose monitoring. Sci Rep (2021) 11(1):23950. doi: 10.1038/s41598-021-03480-9 34907285 PMC8671539

[4] Moser O Riddell MC Eckstein ML Adolfsson P Rabasa-Lhoret R van den Boom L. Glucose management for exercise using continuous glucose monitoring (CGM) and intermittently scanned CGM (isCGM) systems in type 1 diabetes: position statement of the European association for the study of diabetes (EASD) and of the international society for pediatric and adolescent diabetes (ISPAD) endorsed by JDRF and supported by the American diabetes association (ADA). Pediatr Diab (2020) 21(8):1375–93. doi: 10.1111/pedi.13105 PMC7702152 33047481

[5] Zelnick LR Batacchi ZO Ahmad I Dighe A Little RR Trence DL. Continuous glucose monitoring and use of alternative markers to assess glycemia in chronic kidney disease. Diab Care (2020) 43(10):2379. doi: 10.2337/dc20-0915 PMC7510019 32788282

[6] Brown SA Kovatchev BP Raghinaru D Lum JW Buckingham BA Kudva YC. Six-month randomized, multicenter trial of closed-loop control in type 1 diabetes. N Engl J Med (2019) 381(18):1707–17. doi: 10.1056/NEJMoa1907863 PMC7076915 31618560

[7] Bombaci B Passanisi S Alibrandi A D’Arrigo G Patroniti S Averna S. One-year real-world study on comparison among different continuous subcutaneous insulin infusion devices for the management of pediatric patients with type 1 diabetes: the supremacy of hybrid closed-loop systems. Int J Environ Res Public Health (2022) 19(16):10293. doi: 10.3390/ijerph191610293 36011925 PMC9408433

[8] Lombardo F Passanisi S Alibrandi A Bombaci B Bonfanti R Delvecchio M. MiniMed 780G six-month use in children and adolescents with type 1 diabetes: clinical targets and predictors of optimal glucose control. Diab Technol Ther (2023). doi: 10.1089/dia.2022.0491 36763343

[9] Mameli C Smylie GM Galati A Rapone B Cardona-Hernandez R Zuccotti G. Safety, metabolic and psychological outcomes of medtronic MiniMed 670G in children, adolescents and young adults: a systematic review. Eur J Pediatr (2023) 21:1–15. doi: 10.1007/s00431-023-04833-4 PMC9942055 36809498

[10] Castañeda J Mathieu C Aanstoot HJ Arrieta A Da Silva J Shin J. Predictors of time in target glucose range in real-world users of the MiniMed 780G system. Diab Obes Metab (2022) 24(11):2212–21. doi: 10.1111/dom.14807 35791621

[11] Forlenza GP Carlson AL Galindo RJ Kruger DF Levy CJ McGill JB. Real-world evidence supporting tandem control-IQ hybrid closed- loop success in the Medicare and Medicaid type 1 and type 2 diabetes populations. Diabetes Technol Ther (2022) 24(11):814–23. doi: 10.1089/dia.2022.0206 PMC9618372 35763323

